# Therapeutic Oral Application of Carvacrol Alleviates Acute Campylobacteriosis in Mice Harboring a Human Gut Microbiota

**DOI:** 10.3390/biom13020320

**Published:** 2023-02-08

**Authors:** Minnja S. Foote, Ke Du, Soraya Mousavi, Stefan Bereswill, Markus M. Heimesaat

**Affiliations:** Gastrointestinal Microbiology Research Group, Institute of Microbiology, Infectious Diseases and Immunology, Charité-Universitätsmedizin Berlin, Corporate Member of Freie Universität Berlin, Humboldt-Universität zu Berlin and Berlin Institute of Health, 12203 Berlin, Germany

**Keywords:** carvacrol, enteropathogenic infection, *Campylobacter jejuni*, immune-modulatory effects, human-gut-microbiota-associated IL-10^−/−^ mice, campylobacteriosis model, host–pathogen interaction, placebo-controlled preclinical intervention study, One Health concept, food safety

## Abstract

Human *Campylobacter jejuni* infections are rising globally. Since antibiotics are usually not indicated in acute campylobacteriosis, antibiotic-independent intervention measures are desirable. The phenolic compound carvacrol constitutes a promising candidate molecule given its antimicrobial and immune-modulatory features. To test the disease-alleviating effects of oral carvacrol treatment in acute murine campylobacteriosis, IL-10^−/−^ mice harboring a human gut microbiota were perorally infected with *C. jejuni* and treated with carvacrol via the drinking water. Whereas *C. jejuni* stably established in the gastrointestinal tract of mice from the placebo cohort, carvacrol treatment resulted in lower pathogen loads in the small intestines on day 6 post infection. When compared to placebo, carvacrol ameliorated pathogen-induced symptoms including bloody diarrhea that was accompanied by less distinct histopathological and apoptotic cell responses in the colon. Furthermore, innate and adaptive immune cell numbers were lower in the colon of carvacrol- versus placebo-treated mice. Notably, carvacrol application dampened *C. jejuni*-induced secretion of pro-inflammatory mediators in intestinal, extra-intestinal and systemic organs to naive levels and furthermore, resulted in distinct shifts in the fecal microbiota composition. In conclusion, our preclinical placebo-controlled intervention study provides evidence that therapeutic carvacrol application constitutes a promising option to alleviate campylobacteriosis in the infected vertebrate host.

## 1. Introduction

With over 120,000 cases in the European Union in 2020, infections with the enteropathogen *Campylobacter jejuni* are the most frequently reported bacterial cause of foodborne illness and come with significant socioeconomic costs [1]. Enteropathogenic transmission to humans is most commonly linked to the consumption of contaminated food of animal origin such as raw and undercooked meat products from livestock, including poultry, and in other cases, to the ingestion of contaminated surface water and unpasteurized dairy products [2,3,4]. Following gastric and duodenal passage, the motile Gram-negative bacteria adhere to intestinal epithelial cells, migrate into subepithelial tissues, and induce innate as well as adaptive immune responses via Toll-like receptor-4 (TLR-4)-mediated sensing of the bacterial lipo-oligosaccharide (LOS) located on the surface of *C. jejuni* [5,6,7,8]. Immune cells such as neutrophils, macrophages, monocytes, as well as T lymphocytes and B cells infiltrate the colonic mucosa and lamina propria [4,8], causing a release of pro-inflammatory mediators in the intestinal tract, disrupting the epithelial barrier function which in turn, results in an malabsorptive syndrome [9,10].

*C. jejuni*-infected humans complain about gastroenteritis with diverse clinical manifestations, ranging from mild clinical signs to severe symptoms characterized by fever, vomiting, abdominal cramps, and watery or even bloody diarrhea [11,12]. The symptoms usually resolve within two weeks post infection (p.i.), and the majority of patients only need supportive treatment such as fluid and electrolyte replacement and application of analgesics with no indication for antibiotic treatment [4,12]. In other, but rare cases, the intestinal colonization of *C. jejuni* can induce rather severe or even systemic infections, particularly in comorbid and immune-compromised patients, which might require antibiotic therapy [13]. Inadequate use and ineffective control of antibiotic intervention measures in livestock and humans have contributed significantly to the selection of resistant bacterial strains to date [14,15]. *Campylobacter* strains have become increasingly resistant to antibiotics including first-line compounds such as fluoroquinolones and macrolides [14]. The worldwide spread of antibiotic resistance, not only in *Campylobacter,* has imposed serious challenges on global healthcare and food safety, and is now regarded as a critical One Health issue [16]. The One Health concept aims to balance and enhance the interdependent health of humans, animals and ecosystems in order to promote global health and address uprising challenges such as antibiotic resistance and foodborne diseases [17]. According to the One Health approach, multi-sectoral (i.e., humans, animals, and the associated environments) and inter-disciplinary (i.e., human and veterinary medicine) strategies are essential to address antibiotic resistance and their collateral damages [16]. As a consequence, generating pharmaceutical intervention strategies for prophylaxis and treatment of human campylobacteriosis with antibiotic-independent compounds as well as finding alternative measures to control antibiotic resistance not only in the human host but also in the animal reservoirs are appreciable and would contribute to safer food chains and lower pathogen-induced morbidities.

Due to the large biological and structural diversity of their components, plants provide a unique and renewable source of new, efficient, and easily applicable antibacterial compounds [18]. Terpenoids, for instance, constitute plant-derived molecules that have been reported to show potent anti-microbial activity against numerous microorganisms [19,20]. The monoterpenoid carvacrol is present as an essential oil in aromatic plants such as thyme, oregano, and wild bergamot [21]. Numerous studies have evaluated the immune-modulatory, anti-inflammatory, and anti-microbial activities of carvacrol. In vitro studies of the phenolic compound revealed anti-bacterial activities against a multitude of Gram-positive (e.g., *Staphylococcus aureus*, *Listeria monocytogenes*) and Gram-negative (e.g., *Escherichia coli*, *Bacillus cereus*, *Pseudomonas aeruginosa*) bacterial species [22,23,24]. In addition, carvacrol was proven to inhibit biofilm development of several bacterial pathogens including *S. aureus*, *E. coli*, and *C. jejuni*, respectively [25,26,27]. Furthermore, in bacteriostatic concentrations, carvacrol was found to be responsible for damage to bacterial cell walls through the alteration of fatty acid compositions [28]. In vitro studies also showed that in bactericidal concentrations, carvacrol causes the disintegration of the outer membrane and disruption of the cytoplasmic membrane of Gram-negative bacteria [29]. Moreover, the phenolic compound acts as a proton exchanger, reducing the pH gradient across the cytoplasmic membrane, which leads to a failure of the proton-motive force, depletion of the adenosine triphosphate pool, and eventually to cell death [22,30]. According to in vitro and in vivo studies, carvacrol application led to a reduction in *C. jejuni* loads in intestinal samples derived from chicken [31,32] as well as to a reduced virulence gene expression and invasion of *C. jejuni* into chicken cells [33]. Furthermore, application of the synthetic compound caused an inhibition of motility and invasive features of *C. jejuni* in vitro [34]. These findings suggest carvacrol as a promising natural target molecule against *C. jejuni*-induced enterocolitis, but studies addressing the therapeutic effects of carvacrol in acute campylobacteriosis are rather scarce. 

In the past, analyses of *C. jejuni*–host interactions were limited by a lack of reliable in vivo models. Due to their gut microbiota composition, conventional laboratory mice exert a strong colonization resistance against *C. jejuni* [35,36]. Upon microbiota depletion following the application of broad-spectrum antibiotics, secondary abiotic mice can be stably colonized by the pathogen following oral challenge [35,36,37]. However, characteristic clinical signs of human campylobacteriosis mice are lacking in *C. jejuni*-infected secondary abiotic wildtype mice [37]. One needs to take into consideration that rodents are approximately 10,000-fold more resistant against bacterial lipo-polysaccharide (LPS) and LOS expressed on the surface of *C. jejuni* as compared to humans [38]. Given that interleukin-10 (IL-10) is required for resistance against LOS [39], mice can be rendered susceptible to *C. jejuni* LOS by deletion of the murine *il-10* gene [8]. Therefore, secondary abiotic IL-10^−/−^ mice display *C. jejuni*-induced acute enterocolitis within a week p.i. and display severe episodes of human campylobacteriosis [40]. This acute *C. jejuni* infection and inflammation model has been proven to be suitable for testing distinct compounds such as rapamycin (sirolimus) [41], activated charcoal [42], various essential oils [43,44,45], urolithin-A [46], vitamin C [47], and vitamin D [48] regarding their anti-pathogenic and immune-modulatory properties in preclinical intervention studies. 

Previous studies also revealed that host susceptibility for and resistance against distinct enteropathogens including *C. jejuni* [49], *Campylobacter coli* [50], and *Salmonella enterica* [51] are determined by the host-specific intestinal microbiota. In order to elucidate the triangular relationship between enteropathogens on one side and immunity and the human gut microbiota on the other (host) side, we here tested carvacrol in IL-10^−/−^ mice harboring a human gut microbiota by subjecting secondary abiotic mice to oral transplantation of a fecal microbiota from human donors. Human-microbiota-associated mice not only harbor the pathogen in their intestines at high loads, but also present key symptoms of acute campylobacteriosis, suggesting that human fecal microbiota transplantation (hFMT) in mice may be well suited to further examine the molecular mechanisms underlying pathogen–commensal microbiota–host interactions [52]. 

In a recent study, we were able to show that an oral challenge of secondary abiotic IL-10^−/−^ mice with carvacrol in a prophylactic regimen starting four days prior *C. jejuni* infection resulted in lowered intestinal pathogen loads and alleviated symptoms of acute campylobacteriosis [53]. These results prompted us to test whether therapeutic oral carvacrol application to human-gut-microbiota-associated IL-10^−/−^ mice starting two days after infection would exert immune-modulatory and/or anti-*C. jejuni*-directed effects and furthermore, affect the human gut microbiota composition. 

## 2. Materials and Methods

### 2.1. Secondary Abiotic IL-10^−/−^ Mice

**Figure 1:**
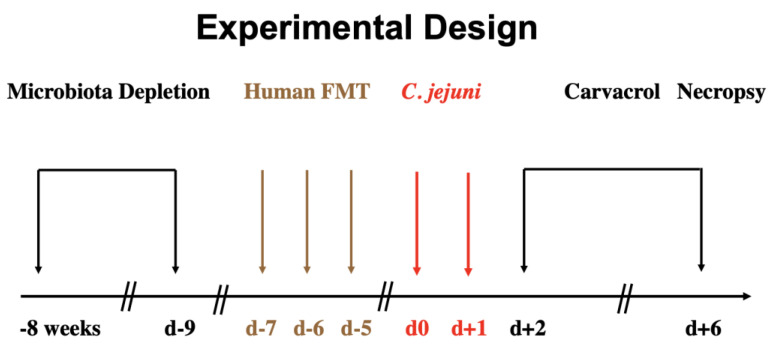
Experimental design. Conventional IL-10^−/−^ mice were treated with ampicillin plus sulbactam via the drinking water for eight weeks in order to deplete the commensal gut microbiota. Two days prior to fecal microbiota transplantation (FMT) from healthy human donors, the antibiotic compound was replaced by autoclaved tap water. Seven days post oral human FMT on day (d) −7, d−6, and d−5, mice were perorally infected with *C. jejuni* on d0 and d+1 and treated with carvacrol via the drinking water from d+2 until the day of necropsy (i.e., d+6).

In the Forschungsinstitute für Experimentelle Medizin, Charité—Universitätsmedizin Berlin, Germany, IL-10^−/−^ C57BL/6j mice were bred and reared under specified pathogen-free (SPF) and standard settings. Mice were housed in autoclaved cages with filter tops within an experimental semi-barrier and had free access to a standard chow diet (food pellets: ssniff R/M-H, V1534-300, Sniff, Soest, Germany) and autoclaved tap water. For a successful induction of intestinal *C. jejuni* colonization, 3-week-old female and male mice were subjected to an antibiotic regimen of ampicillin plus sulbactam (2 g/L; Dr. Friedrich Eberth Arzneimittel, Ursensollen, Germany) via the drinking water for eight weeks (ad libitum) in order to eradicate the murine intestinal microbiota (Figure 1) as described recently [54]. To ensure effective microbiota depletion, fecal samples were cultivated in enrichment broths as reported in detail [55]. All secondary abiotic mice were maintained and handled under aseptic conditions to avoid cross-contamination. Two days before hFMT the antibiotic treatment was withdrawn (Figure 1) and replaced by autoclaved water (ad libitum).

### 2.2. Human Fecal Microbiota Transplantation 

In order to introduce a complex human intestinal microbiota into the murine host, the secondary abiotic mice received hFMT starting 1 week prior to *C. jejuni* infection on three consecutive days (i.e., on days −7, −6, and −5; Figure 1) as described in detail previously [37,52,56]. Therefore, human fecal samples collected from 5 healthy individuals (all samples free of enteropathogenic bacteria, viruses, and parasites) and aliquoted and stored at −80 °C were thawed, resuspended in sterile phosphate buffered saline (PBS, Thermo Fisher Scientific, Waltham, MA, USA), and pooled before oral application to mice via gavage (0.3 mL volume). The microbiota composition of the suspensions used for hFMT is shown in Supplemental Figure S1.

### 2.3. Campylobacter Jejuni Infection

Viable *C. jejuni* strain 81-176 bacteria stored at −80 °C were thawed, streaked out, and incubated on selective karmali agar plates (purchased from Oxoid, Wesel, Germany) at 37 °C for 48 h under micro-aerophilic conditions. In order to generate an inoculum of 10^9^ bacterial cells, bacteria were harvested in sterile PBS (Thermo Fisher Scientific, Waltham, MA, USA). Age- and sex-matched human-gut-microbiota-associated IL-10^−/−^ mice (3-month-old littermates) were then infected perorally with 10^9^ colony forming units (CFU) of the pathogen on two consecutive days (i.e., on days 0 and 1; Figure 1) by gavage as stated in detail elsewhere [57].

### 2.4. Treatment Regimen

Oral carvacrol treatment via the drinking water was initiated two days after initial *C. jejuni* infection. Therefore, synthetic carvacrol (purchased from Sigma-Aldrich, Munich, Germany) was dissolved in Tween 80 (0.2% *v/v*) and added into autoclaved tap water to a final concentration of 500 mg/L (ad libitum) resulting in a daily dose of 100 mg per kg of body weight considering a body weight of approximately 25 g per mouse and a daily drinking volume of approximately 5 mL. The placebo control animals received a vehicle via the drinking water instead. Treatment solutions were changed every other day until the end of the experimental period (i.e., day 6 p.i.; Figure 1).

### 2.5. Gastrointestinal C. jejuni Loads

For the determination of gastrointestinal pathogen loads, the numbers of live *C. jejuni* bacteria after oral infection by gavage were monitored in fecal samples daily and upon necropsy in intraluminal gastrointestinal samples taken from the stomach, duodenum, ileum, and colon lumen that were subsequently homogenized in PBS (Thermo Fisher Scientific, Waltham, MA, USA). *C. jejuni* was quantified by the counting of CFU after the growth of serial dilutions of intestinal samples on karmali agar for at least 48 h at 37 °C under micro-aerophilic conditions as described in detail previously [54]. The detection limit of viable pathogens was 100 CFU per g of fecal sample.

### 2.6. Gut Microbiota Composition

The microbiota composition of human fecal donor suspensions used for hFMT and of murine fecal samples of human-microbiota-associated mice was analyzed immediately before (i.e., day 0) and 6 days after *C. jejuni* infection as described in detail previously [55,58,59]. In brief, for cultural analyses, respective samples were homogenized in sterile PBS (Thermo Fisher Scientific, Waltham, MA, USA) and serial dilutions were streaked onto solid culture media and incubated under aerobic, micro-aerobic and anaerobic conditions for at least 48 h. Total bacterial loads and individual bacteria were enumerated and after subcultivation, species were identified according to their colony morphology, Gram-staining, biochemical analysis, and sequencing [55]. In addition, culture-independent 16s-rRNA-based methods were applied to confirm cultural results and furthermore, to quantitatively assess fastidious and even non-cultivable bacteria. Therefore, the total genomic DNA was extracted from respective samples and the main bacterial groups that are abundant in the human gut microbiota were determined by quantitative real-time polymerase chain reaction applying species-, genera- or group-specific 16S rRNA primers (Tib MolBiol, Berlin, Germany) and expressed as gene copies per ng of DNA [58,59].

### 2.7. Clinical Conditions of Mice

Immediately before and every day after *C. jejuni* infection, the clinical outcome of the mice was monitored quantitatively by using a cumulative clinical score (maximum 12 points), addressing the clinical aspects of the animals (i.e., wasting symptoms; 0: normal; 1: ruffled fur; 2: less locomotion; 3: isolation; 4: severely compromised locomotion, pre-final aspect), the occurrence of fecal blood (0: no blood; 2: microscopic detection of blood by the Guajac method using Haemoccult, Beckman Coulter/PCD, Germany; 4: macroscopic blood visible), and the stool consistency (0: formed feces; 2: pasty feces; 4: liquid feces), as stated elsewhere [60].

### 2.8. Sampling Procedures

At day 6 p.i., mice were sacrificed by carbon dioxide asphyxiation. Cardiac blood for cytokine measurements and ex vivo biopsies from liver, kidneys, mesenteric lymph nodes (MLN), and colon as well as luminal samples from stomach, duodenum, ileum, and colon were removed under aseptic conditions for microbiological, immunological, and immunohistopathological analyses.

### 2.9. Histopathology

For histopathological analyses, colon ex vivo biopsies were immediately fixed in 5% formalin, embedded in paraffin and 5 µm sections stained with hematoxylin and eosin (H&E). In order to evaluate the severity of histopathological changes of the colonic mucosa, respective biopsies were assessed by light microscopy (100 × magnification) and quantitated by applying an established scoring scheme [61]: Score 1, minimal inflammatory cell infiltrates in the mucosa with intact epithelium; score 2, mild inflammatory cell infiltrates in the mucosa and submucosa with mild hyperplasia and mild goblet cell loss; score 3, moderate inflammatory cell infiltrates in the mucosa with moderate goblet cell loss; score 4, marked inflammatory cell infiltration into the mucosa and submucosa with marked goblet cell loss, multiple crypt abscesses, and crypt loss.

### 2.10. In Situ Immunohistochemistry

For in situ immunohistochemical analyses, colonic ex vivo biopsies were fixed in 5% formalin and embedded in paraffin as recently reported [62]. In brief, an independent investigator counted numbers of specifically stained cells by light microscopy in order to detect apoptotic epithelial cells, neutrophils, macrophages and monocytes, T lymphocytes, and regulatory T cells. Respective cells were counted in colonic paraffin sections (5 µm) stained with primary antibodies against cleaved caspase-3 (Asp175, Cell Signaling, Beverly, MA, USA, 1:200), MPO7 (No. A0398, Dako, Glostrup, Denmark, 1:500), F4/80 (no. 14-4801, clone BM8, eBioscience, San Diego, CA, USA, 1:50), CD3 (no. N1580, Dako, 1:10), and FOXP3 (clone FJK-165, no. 14-5773, eBioscience, 1:100), respectively. The mean number of positively stained cells in each blinded sample was determined within at least six high-power fields (HPF, 0.287 mm^2^, 400 × magnification).

### 2.11. Pro-Inflammatory Mediators

Colonic samples (approximately 1 cm^2^) that were longitudinally cut and washed in sterile PBS and ex vivo biopsies derived from MLN (3 nodes), the liver (approximately 1 cm^3^), and the kidney (one half after the longitudinal cut) were transferred to 24 flat-bottom well culture plates (Thermo Fisher Scientific, Waltham, MA, USA) containing 500 µL serum-free RPMI 1640 medium (Thermo Fisher Scientific, Waltham, MA, USA), penicillin (100 µg/mL; Biochrom, Berlin, Germany), and streptomycin (100 µg/mL; Biochrom, Berlin, Germany). After an 18 h incubation period at 37 °C, respective culture supernatants and serum samples were tested for interferon-γ (IFN-γ) and interleukin-6 (IL-6) by the Mouse Inflammation Cytometric Bead Assay (BD Biosciences, Germany) in a BD FACSCanto II flow cytometer (BD Biosciences). Nitric oxide concentrations were determined with the Griess reaction [63].

### 2.12. Statistical Analyses

After pooling of data from three independent experiments, medians and significance levels were calculated using GraphPad Prism (version 9; San Diego, CA, USA). Applying the Anderson–Darling test, the normalization of data sets was surveyed. The Student’s t-test and Mann–Whitney test were used for pairwise comparisons of normally and not-normally distributed data, respectively. Multiple comparisons were performed using the one-way ANOVA with Tukey post-correction (for normally distributed data) and Kruskal–Wallis test with Dunn’s post-correction (for not-normally distributed data). Two-sided probability (*p*) values ≤ 0.05 were considered significant.

## 3. Results

### 3.1. Gastrointestinal C. jejuni Colonization upon Carvacrol Treatment of Infected IL-10^−/−^ Mice with a Human Gut Microbiota

**Figure 2:**
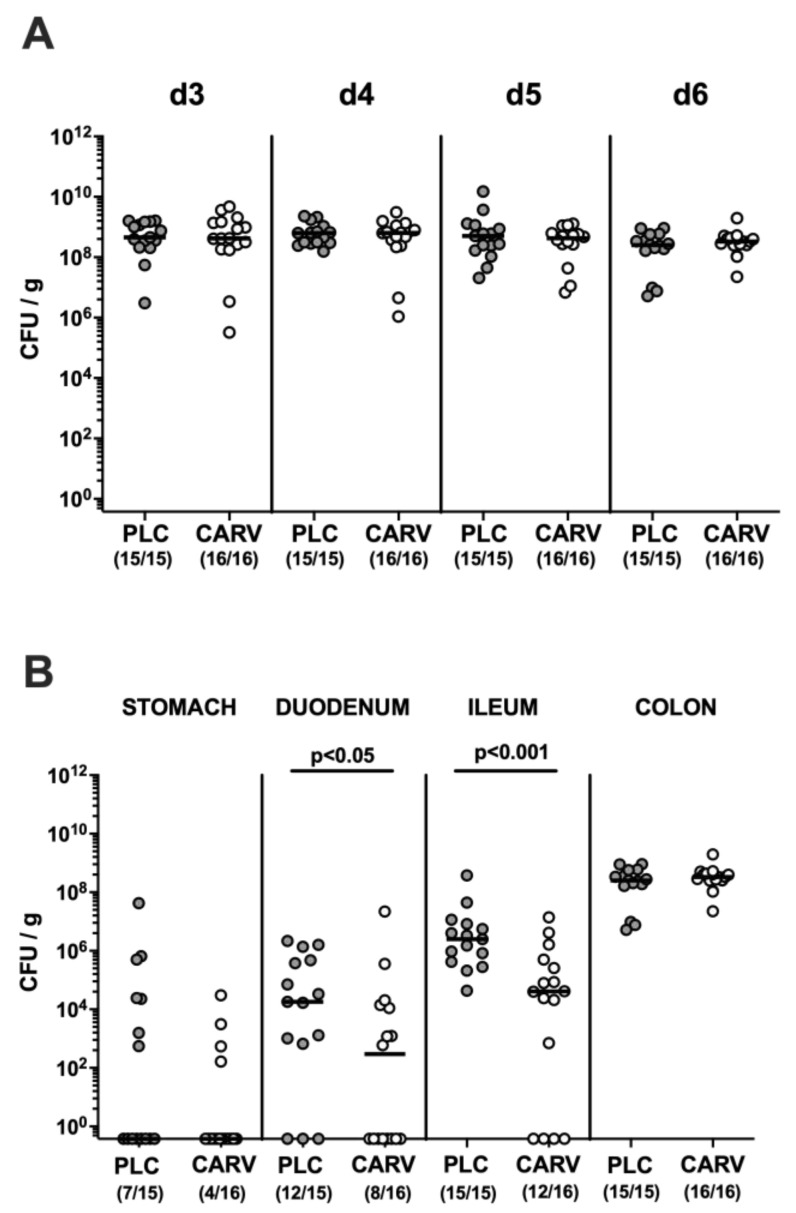
Gastrointestinal *C. jejuni* colonization upon carvacrol treatment of infected IL-10^−/−^ mice with a human gut microbiota. IL-10^−/−^ mice harboring a human gut microbiota were perorally infected with *C. jejuni* strain 81-176 on day (d)0 and d1. Starting on d2 post infection (p.i.), mice were treated with either synthetic carvacrol (CARV, white circles) or placebo (PLC, grey circles) via the drinking water until d6 p.i. (**A**) Fecal *C. jejuni* loads were determined by culture at defined time points p.i. as indicated. (**B**) Upon sacrifice on d6 p.i., *C. jejuni* were cultured from distinct gastrointestinal locations. Data pooled from three experiments, medians, numbers of culture-positive mice out of the total cohort (in parentheses), and significance levels (*p* values) determined by the Mann–Whitney test are shown.

We first addressed whether *C. jejuni* could stably establish in the intestinal tract of human-gut-microbiota-associated IL-10^−/−^ mice following oral challenge and furthermore, whether carvacrol treatment starting on day 2 p.i. (Figure 1) would impact gastrointestinal pathogen loads. Our cultural analyses of fecal samples over time revealed comparably high *C. jejuni* cell numbers in both carvacrol- and placebo-treated mice from day 3 until day 6 p.i. (not significant (n.s.); Figure 2A). On the day of necropsy (i.e., day 6 p.i.) we further assessed enteropathogen loads alongside the gastrointestinal tract and detected approximately two orders of magnitude lower median luminal *C. jejuni* counts in proximal and distal parts of the small intestines such as the duodenum and terminal ileum, respectively, following carvacrol as compared to placebo treatment (*p* < 0.05–0.001; Figure 2B). In the stomach and the colon, however, the *C. jejuni* numbers were comparable on day 6 p.i. (n.s.; Figure 2B). Hence, *C. jejuni* could stably establish in the intestinal tract of infected IL-10^−/−^ mice harboring a human gut microbiota, whereas carvacrol treatment resulted in lower enteropathogen loads in the small intestines.

### 3.2. Clinical Conditions over Time upon Carvacrol Treatment of Infected IL-10^−/−^ Mice with a Human Gut Microbiota

**Figure 3:**
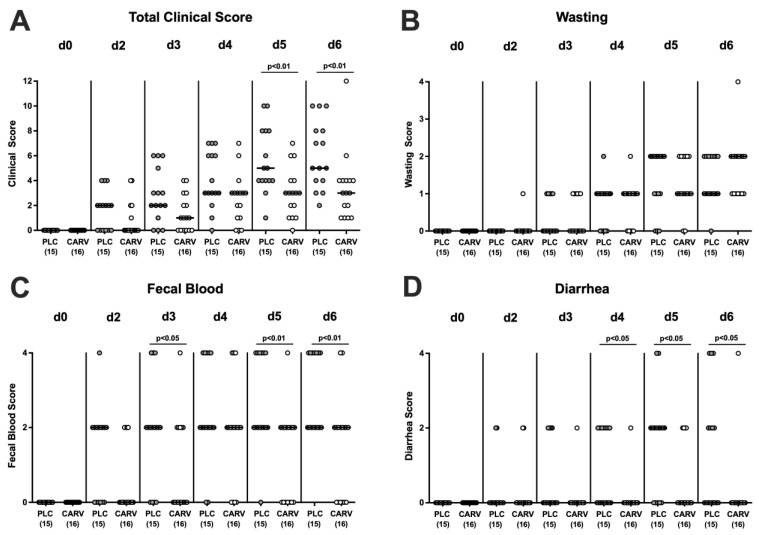
Clinical conditions over time upon carvacrol treatment of infected IL-10^−/−^ mice with a human gut microbiota. IL-10^−/−^ mice harboring a human gut microbiota were perorally infected with *C. jejuni* on day (d)0 and d1. Starting on d2 post infection (p.i.), mice were treated with either synthetic carvacrol (CARV, white circles) or placebo (PLC, grey circles) via the drinking water until d6 p.i. The clinical conditions of mice were monitored and quantitated at defined time points p.i. by a clinical scoring scheme and expressed as (**A**) total clinical scores constituting the sum of the scores for (**B**) wasting symptoms, (**C**) fecal blood, and (**D**) diarrheal symptoms. Data pooled from three experiments, medians, numbers of included mice (in parentheses), and significance levels (*p* values) as determined by the Mann–Whitney test and the Student’s *t*-test are shown.

We further surveyed the clinical signs of *C. jejuni*-induced disease such as wasting symptoms, stool consistency, and fecal blood following carvacrol treatment and applied a defined scoring scheme. As early as three days following the initiation of the oral challenge (i.e., day 5 p.i.), carvacrol-treated mice displayed lower total clinical scores when compared to their placebo counterparts (*p* < 0.01; Figure 3A). Whereas scores assessing wasting symptoms did not differ between respective cohorts (n.s.; Figure 3B), mice from the carvacrol treatment group exhibited lower scores for the abundance of blood in fecal samples when compared to placebo controls on days 3, 5, and 6 p.i. (*p* < 0.05–0.01; Figure 3C), which also held true for diarrheal scores on days 4, 5, and 6 p.i. (*p* < 0.05; Figure 3D). Hence, carvacrol treatment of *C. jejuni*-infected mice ameliorated pathogen-induced symptoms such as bloody diarrhea. 

### 3.3. Microscopic Inflammatory Changes in the Colon upon Carvacrol Treatment of Infected IL-10^−/−^ Mice with a Human Gut Microbiota

**Figure 4:**
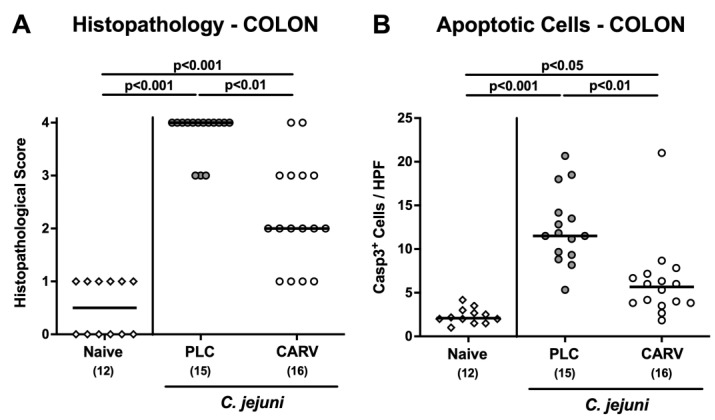
Microscopic inflammatory changes in the colon upon carvacrol treatment of infected IL-10^−/−^ mice with a human gut microbiota. IL-10^−/−^ mice harboring a human gut microbiota were perorally infected with *C. jejuni* on day (d)0 and d1. Starting on d2 post infection (p.i.), mice were treated with either synthetic carvacrol (CARV, white circles) or placebo (PLC, grey circles) via the drinking water until d6 p.i. Naive mice served as uninfected and untreated controls (white diamonds). Upon sacrifice on d6 p.i., (**A**) histopathological changes were quantitated by histopathological scores. Moreover, (**B**) apoptotic epithelial cells were enumerated in caspase3-positive (Casp3^+^) colonic paraffin sections by light microscopy and the average numbers of positively stained cells out of six representative high-power fields (HPF, 400 × magnification) were determined. Data pooled from three experiments, medians, numbers of included mice (in parentheses), and significance levels (*p* values) as determined by the Kruskal–Wallis test with Dunn’s post-correction are shown.

We next addressed whether the better macroscopic, i.e., clinical outcome upon carvacrol treatment was accompanied by less severe microscopic inflammatory sequelae of *C. jejuni* infection. Therefore, we quantitated histopathological changes in the colon by using a histopathological scoring scheme. On day 6 p.i., carvacrol-treated mice displayed less severe pathogen-induced histopathological cell damage in the colon when compared to placebo control animals as indicated by lower histopathological sores in the former versus the latter (*p* < 0.01; Figure 4A). In addition, we assessed the extent of *C. jejuni*-induced apoptotic cell responses in the colon of mice from respective treatment regimens on day 6 p.i. and found lower numbers of epithelial cells that were positive for cleaved caspase-3 in colon samples taken from carvacrol as compared to placebo-treated mice (*p* < 0.01; Figure 4B). Hence, carvacrol treatment ameliorated histopathological and apoptotic cell responses in the colon of *C. jejuni*-infected mice. 

### 3.4. Immune Cell Responses in the Colon upon Carvacrol Treatment of Infected IL-10^−/−^ Mice Harboring a Human Gut Microbiota

**Figure 5:**
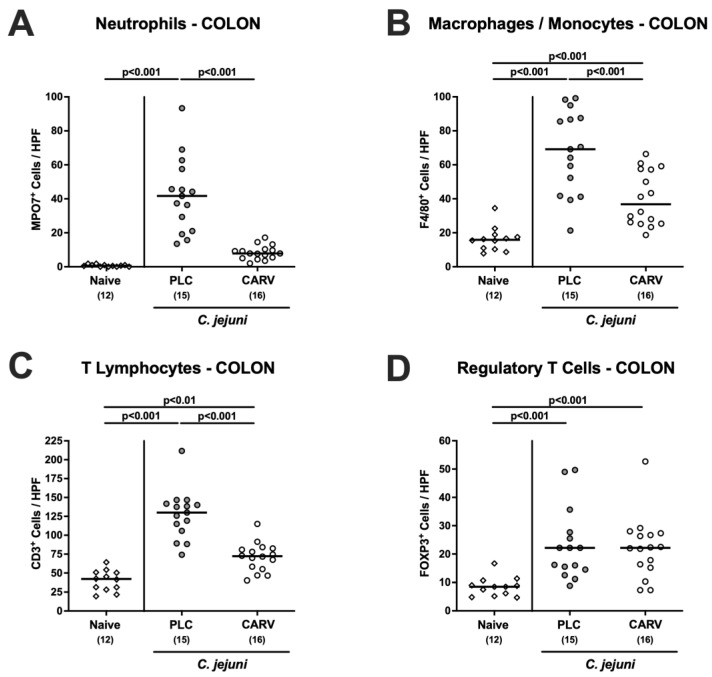
Immune cell responses in the colon upon carvacrol treatment of infected IL-10^−/−^ mice harboring a human gut microbiota. IL-10^−/−^ mice harboring a human gut microbiota were perorally infected with *C. jejuni* on day (d)0 and d1. Starting on d2 post infection (p.i.), mice were treated with either synthetic carvacrol (CARV, white circles) or placebo (PLC, grey circles) via the drinking water until d6 p.i. Naive mice served as uninfected and untreated controls (white diamonds). Upon sacrifice on d6 p.i., distinct immune cell populations such as (**A**) neutrophils (MPO7^+^), (**B**) macrophages and monocytes (F4/80^+^), (**C**) T lymphocytes (CD3^+^), and (**D**) regulatory T cells (FOXP3^+^) were enumerated in colonic paraffin sections by light microscopy and the average numbers of positively stained cells out of six representative high-power fields (HPF, 400 × magnification) were determined. Data pooled from three experiments, medians, numbers of included mice (in parentheses), and significance levels (*p* values) as determined by the one-way ANOVA test with Tukey post-correction and the Kruskal–Wallis test with Dunn’s post-correction are shown.

We then tested the impact of carvacrol treatment on *C. jejuni*-induced innate and adaptive immune responses in the colon. Our quantitative in situ immunohistochemical analyses revealed that numbers of innate immune cell subsets such as MPO7^+^ neutrophils and of F4/80^+^ macrophages and monocytes were lower in the colonic mucosa and lamina propria of carvacrol- as compared to placebo-treated mice on day 6 p.i. (*p* < 0.001; Figure 5A,B). Of note, colonic neutrophil numbers in carvacrol-treated infected mice did not differ from those found in naive animals (n.s.; Figure 5A). Furthermore, *C. jejuni* infection was accompanied by increases in adaptive immune cell populations including CD3^+^ T lymphocytes and FOXP3^+^ regulatory T cells (*p* < 0.01–0.001 versus naive; Figure 5C,D). The pathogen-induced increases in colonic T cell numbers were, however, less pronounced following carvacrol as compared to placebo treatment (*p* < 0.001; Figure 5C). Hence, carvacrol treatment resulted in less distinct innate and adaptive immune cell responses in the colon of *C. jejuni*-infected mice.

### 3.5. Intestinal Pro-Inflammatory Mediator Secretion upon Carvacrol Treatment of Infected IL-10^−/−^ Mice Harboring a Human Gut Microbiota

**Figure 6:**
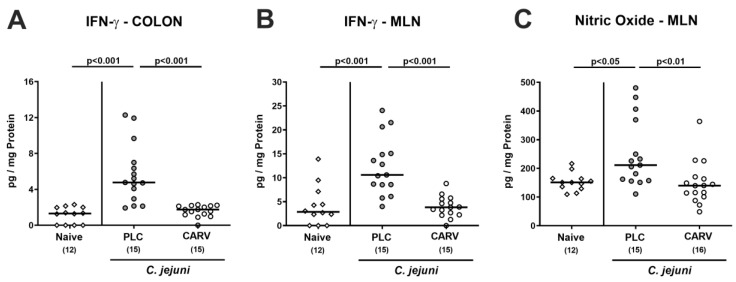
Intestinal pro-inflammatory mediator secretion upon carvacrol treatment of infected IL-10^−/−^ mice harboring a human gut microbiota. IL-10^−/−^ mice harboring a human gut microbiota were perorally infected with *C. jejuni* on day (d)0 and d1. Starting on d2 post infection (p.i.), mice were treated with either synthetic carvacrol (CARV, white circles) or placebo (PLC, grey circles) via the drinking water until d6 p.i. Naive mice served as uninfected and untreated controls (white diamonds). Upon sacrifice on d6 p.i., IFN-γ concentrations were measured in ex vivo biopsies taken from the (**A**) colon and (**B**) mesenteric lymph nodes (MLN) and furthermore, (**C**) nitric oxide secretion was assessed in MLN. Data pooled from three experiments, medians, numbers of included mice (in parentheses), and significance levels (*p* values) as determined by the Kruskal–Wallis test with Dunn’s post-correction are shown. Definite outliers were removed after being identified by the Grubb’s test (α = 0.05).

We next tested the effects of carvacrol treatment on *C. jejuni*-induced pro-inflammatory mediator secretion in distinct intestinal compartments. On day 6 p.i., increased IFN-γ concentrations could be determined in ex vivo biopsies taken from the colon and MLN of placebo-, but not of carvacrol-treated mice, which also held true for nitric oxide levels measured in MLN (*p* < 0.05–0.001 versus naive; Figure 6). Hence, carvacrol treatment dampened *C. jejuni*-induced pro-inflammatory mediator responses in the intestinal tract to naive levels. 

### 3.6. Extra-Intestinal and Systemic Pro-Inflammatory Cytokine Secretion upon Carvacrol Treatment of Infected IL-10^−/−^ Mice Harboring a Human Gut Microbiota

**Figure 7:**
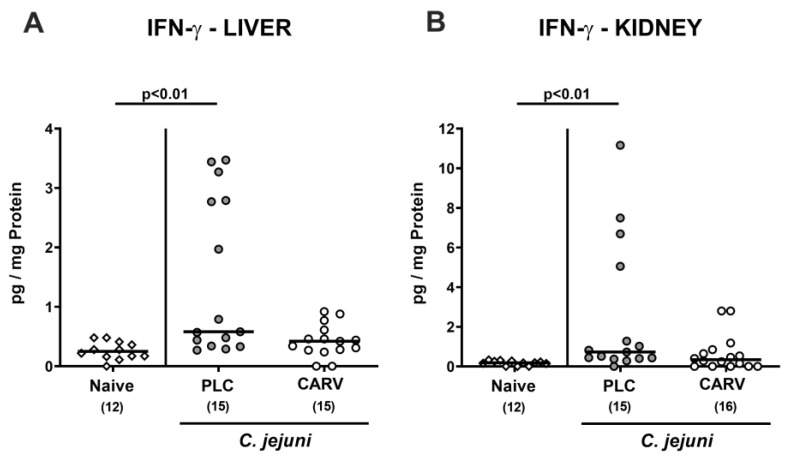
Extra-intestinal and systemic pro-inflammatory cytokine secretion upon carvacrol treatment of infected IL-10^−/−^ mice harboring a human gut microbiota. IL-10^−/−^ mice harboring a human gut microbiota were perorally infected with *C. jejuni* on day (d)0 and d1. Starting on d2 post infection (p.i.), mice were treated with either synthetic carvacrol (CARV, white circles) or placebo (PLC, grey circles) via the drinking water until d6 p.i. Naive mice served as uninfected and untreated controls (white diamonds). Upon sacrifice on d6 p.i., IFN-γ concentrations were measured in ex vivo biopsies taken from the (**A**) liver and (**B**) kidneys. Data pooled from three experiments, medians, numbers of included mice (in parentheses), and significance levels (*p* values) by the Kruskal–Wallis test with Dunn’s post-correction are shown. A definite outlier was removed after being identified by the Grubb’s test (α = 0.05).

We further asked whether the inflammation-dampening effect of carvacrol was restricted to the intestinal tract or was also effective at extra-intestinal locations. In fact, increased IFN-γ concentrations could exclusively be measured in liver and kidney samples taken from placebo treated mice on day 6 p.i. (*p* < 0.01 versus naive; Figure 7), whereas respective cytokine concentrations did not differ in carvacrol-treated infected mice and naive counterparts (n.s.; Figure 7). 

**Figure 8:**
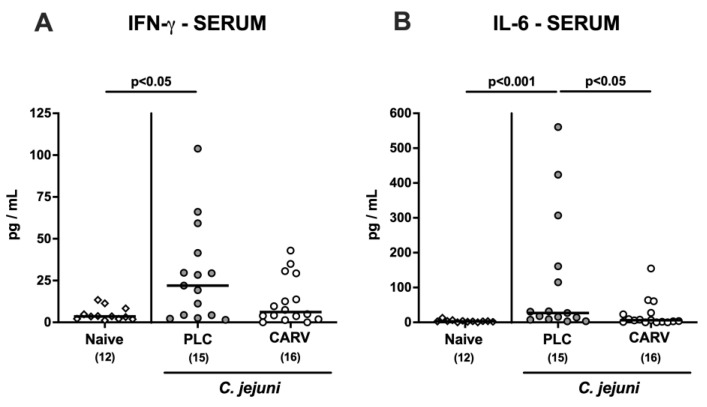
Systemic pro-inflammatory cytokine secretion upon carvacrol treatment of infected IL-10^−/−^ mice harboring a human gut microbiota. IL-10^−/−^ mice harboring a human gut microbiota were perorally infected with *C. jejuni* on day (d)0 and d1. Starting on d2 post infection (p.i.), mice were treated with either synthetic carvacrol (CARV, white circles) or placebo (PLC, grey circles) via the drinking water until d6 p.i. Naive mice served as uninfected and untreated controls (white diamonds). Upon sacrifice on d6 p.i., (**A**) IFN-γ and (**B**) IL-6 concentrations were measured in serum samples. Data pooled from three experiments, medians, numbers of included mice (in parentheses), and significance levels (*p* values) determined by the Kruskal–Wallis test with Dunn’s post-correction are shown.

Strikingly, carvacrol also dampened the systemic pathogen-induced pro-inflammatory cytokine responses as indicated by IFN-γ and IL-6 concentrations, which were increased in serum samples derived from placebo-, but not carvacrol-treated mice on day 6 p.i. (*p* < 0.05 and *p* < 0.001 versus naive, respectively; Figure 8). Hence, therapeutic carvacrol application to *C. jejuni*-infected mice dampened not only intestinal, but also extra-intestinal and even systemic pathogen-induced pro-inflammatory cytokine responses.

### 3.7. Impact of Carvacrol Treatment on Fecal Microbiota Composition of Infected IL-10^−/−^ Mice Harboring a Human Gut Microbiota

**Figure 9:**
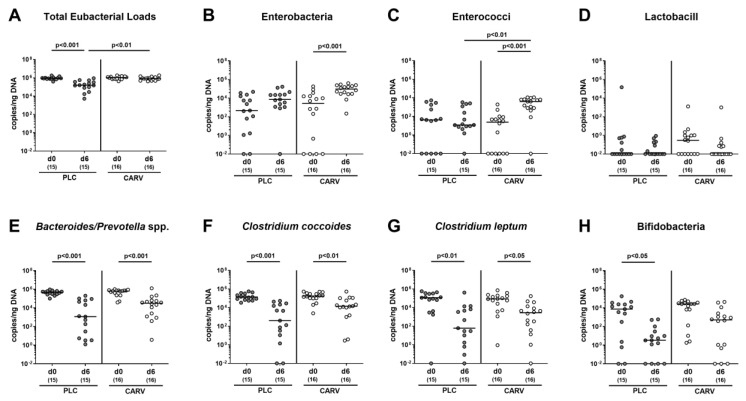
Fecal microbiota composition changes upon carvacrol treatment of infected IL-10^−/−^ mice harboring a human gut microbiota. IL-10^−/−^ mice harboring a human gut microbiota were perorally infected with *C. jejuni* on day (d)0 and d1. Starting on d2 post infection (p.i.), mice were treated with either synthetic carvacrol (CARV, white circles) or placebo (PLC, grey circles) via the drinking water until d6 p.i. Before d0 and upon sacrifice 6 days after infection, the fecal microbiota composition was quantitatively surveyed applying culture-independent molecular methods, and the respective bacterial numbers were expressed as gene copies per ng of DNA. Data pooled from three experiments, medians, numbers of included mice (in parentheses), and significance levels (*p* values) as determined by the Kruskal–Wallis test with Dunn’s post-correction are shown.

We finally addressed whether the gut microbiota of infected mice harboring a human gut microbiota differed upon carvacrol and placebo treatment during *C. jejuni* infection. Therefore, we performed a comprehensive gut microbiota analysis of fecal samples applying cultural as well as culture-independent analyses and found that immediately before *C. jejuni* infection (i.e., on day 0), the fecal microbiota composition did not differ between mice from both cohorts (n.s.; Supplemental Figure S2 and Figure 9). In the course of *C. jejuni* infection, however, the total bacterial and bifidobacterial loads decreased in fecal samples taken from placebo- as opposed to carvacrol-treated mice (*p* < 0.001; Supplemental Figure S2A; *p* < 0.001 and *p* < 0.05, respectively; Figure 9A,H). Furthermore, lower fecal *Bacteroides/Prevotella* species and *Clostridium/Eubacterium* species including *Clostridium coccoides* and *Clostridium leptum* groups could be detected in mice from both cohorts on day 6 as compared to day 0 (*p* < 0.05–0.001; Supplemental Figure S2E,F and Figure 9E–G). Conversely, during *C. jejuni* infection, intestinal loads of enterobacteria and enterococci increased in carvacrol-, but not placebo-treated mice (*p* < 0.05–0.001; Supplemental Figure S2B,C and Figure 9B,C). At the end of the experiment, mice from the carvacrol cohort exhibited slightly higher total eubacterial bacterial loads in their feces when compared to their placebo counterparts (*p* < 0.01; Figure 9A), which also held true for higher bacterial cell counts and gene numbers of enterococci in the feces derived from the former versus the latter on day 6 p.i. (*p* < 0.01; Supplemental Figure S2C and Figure 9C). Hence, carvacrol treatment of *C. jejuni*-infected mice harboring a human gut microbiota was associated with distinct shifts in the fecal microbiota composition.

## 4. Discussion

In our present placebo-controlled intervention study, we tested the anti-pathogenic and immune-modulatory effects of synthetic carvacrol upon oral therapeutic application to *C. jejuni*-infected IL-10^−/−^ mice harboring a human gut microbiota. Following carvacrol treatment starting two days p.i., human-gut-microbiota-associated IL-10^−/−^ mice harbored up to two orders of magnitude lower *C. jejuni* loads in both the proximal and distal parts of the small intestines (i.e., the duodenum and ileum, respectively) compared to placebo control mice. In the colon, however, the *C. jejuni* numbers were comparable in mice from both cohorts. This is to some extent surprising given that the concentration of carvacrol applied via the drinking water was 500 mg/L, which is more than three times higher than the minimal inhibitory concentration (MIC) of 150 mg/L when tested against *C. jejuni* strain 81-176 in vitro [53]. One explanation could be that most of the compound was reabsorbed by enterocytes before reaching the colonic lumen. Furthermore, dilutional effects by intestinal fluids might have resulted in less pronounced anti-*C. jejuni*-directed effects. Interestingly, in our recent study, prophylactic application of carvacrol to secondary abiotic IL-10^−/−^ mice resulted in lower pathogen numbers in the ileum and colon, but neither in the stomach, nor the duodenum [53]. The abundance of the human gut microbiota leading to a different intraluminal milieu might be an additional factor explaining the different gastrointestinal *C. jejuni* loads.

Whereas placebo-treated human-gut-microbiota-associated IL-10^−/−^ mice were suffering from severe campylobacteriosis, carvacrol treatment resulted in improved clinical conditions as early as 5 days p.i., particularly in less severe bloody diarrhea despite the high pathogenic burdens in the large intestines. It is tempting to speculate that these disease-alleviating effects might have been—at least in part—due to the down-regulatory effects of carvacrol on the expression of pathogenic virulence genes involved in motility, adhesion, and invasion as shown in several in vitro studies [25,33,64,65]. The improved clinical outcome was accompanied by less distinct microscopic inflammatory signs of *C. jejuni* infection such as histopathological changes of the large intestinal mucosa and colonic epithelial cell apoptosis. In line with these findings, a recent in vitro study revealed potent anti-apoptotic effects of synthetic carvacrol, given the reduced expression of the apoptosis-inducing factor (AIF) upon treatment of cadmium-stimulated cells resulting in suppressed cleavage of caspase-3 [66]. Furthermore, carvacrol application could effectively down regulate the mammalian target of rapamycin (mTOR) [66]. Interestingly, mTOR is the cellular target for the immune suppressor rapamycin (sirolimus) that has been shown to potently ameliorate acute campylobacteriosis in infected IL-10^−/−^ mice [41]. Our results are supported by our previous study showing that prophylactic carvacrol treatment alleviated *C. jejuni*-induced symptoms of campylobacteriosis including wasting and bloody diarrhea as early as day 3 p.i. and moreover, reduced distinct *C. jejuni*-induced apoptosis in colonic epithelia [53]. Despite the shorter treatment period in our study, carvacrol could still effectively improve clinical outcomes upon *C. jejuni* infection. 

Moreover, carvacrol treatment of *C. jejuni*-infected mice was accompanied by less distinct pro-inflammatory immune responses as indicated by less accumulation of innate and adaptive immune cell subsets such as macrophages, monocytes, neutrophils, and T lymphocytes in the colonic mucosa. Notably, intestinal concentrations of pro-inflammatory mediators such as IFN-γ and nitric oxide measured in carvacrol-treated, *C. jejuni*-infected mice were comparable to those detected in uninfected counterparts. These dampened pro-inflammatory immune responses resulted in diminishing oxidative stress to the intestinal epithelium, less pronounced histopathological and apoptotic colonic epithelial changes, and alleviated enteritis symptoms. In support of this, recent in vitro studies revealed that exogenous carvacrol exerted enhanced radical-scavenging activities [67] and led to diminished nitric oxide and IFN-γ secretion from stimulated macrophages and T lymphocytes, respectively [68,69]. Furthermore, the oral administration of carvacrol to LPS-challenged broiler chickens resulted in down-regulated intestinal expression of pro-inflammatory cytokines [70]. Given that *C. jejuni*-induced enterocolitis is mainly due to TLR-4-mediated sensing of the pathogenic LOS, the TLR-4 antagonistic effects of carvacrol constitutes an important molecular mechanism underlying the disease-alleviating effects of the phenolic compound in campylobacteriosis. The anti-TLR-4 directed actions of carvacrol have been further demonstrated in various tissues such as stimulated cardiomyocytes [71], rheumatoid-arthritis-induced fibroblast-like synoviocytes [72], vascular endothelial cells [73], and brain neuronal cells [74].

Remarkably, the anti-inflammatory effects of exogenous carvacrol were not only restricted to the intestinal tract, but also affected extra-intestinal and even systemic tissue sites as indicated by attenuated secretion of cytokines such as IFN-γ and IL-6 in liver, kidney, and serum samples taken from carvacrol-treated, infected mice. These results are in line with the findings of our recent study applying carvacrol to secondary abiotic IL-10^−/−^ mice prior to *C. jejuni* infection [53]. Furthermore, carvacrol treatment of rats subjected to cadmium toxicity could alleviate oxidative stress and inflammation including apoptotic responses in affected liver and kidney tissues [75]. Of note, oral carvacrol application protected diabetic mice from liver injuries by down regulating not only the TLR-4 but also the mTOR signaling pathway [73]. The systemic anti-inflammatory effects of exogenous carvacrol observed in our current and previous study [53] are further underscored by a better survival of carvacrol-treated mice suffering from acute lung injury and LPS-induced endotoxinemia that was accompanied by diminished concentrations of IL-6 and myeloperoxidase derived from neutrophilic granulocytes [76].

The human-gut-microbiota-associated IL-10^−/−^ mice applied in this study have been shown to present a reliable *C. jejuni* infection and inflammation model to dissect the interactions between the enteropathogen on one side, and of immunity and human gut microbiota on the host side [52]. As for every model, limitations must be taken into considerations. For instance, some species of the human microbiota might have been reduced or lost upon freezing, thawing, or further processing of the fecal donor samples [56]. Additionally, environmental factors such as housing conditions, diet, genetic background, and immunological status may impact the composition of the host-specific gut microbiota [56,77,78]. 

As confirmed by both culture and culture-independent analyses of fecal samples immediately before *C. jejuni* infection (i.e., on day 0), the gut microbiota compositions were comparable in mice from the carvacrol and the placebo cohorts, indicative of similarly effective engraftments following human FMT. At day 6 p.i., however, carvacrol-treated mice presented with slightly different fecal microbiota signatures characterized by higher numbers of total bacterial loads and enterococci when compared to their placebo counterparts. Furthermore, in the course of *C. jejuni* infection, the placebo- as opposed to carvacrol-treated mice experienced a significant (i.e., approximately 4 orders of magnitude) drop in fecal bifidobacteria from day 0 until day 6 p.i. In particular, the bifidobacterial species are known as important members of the commensal gut microbiota contributing to intestinal homeostasis due to short chain fatty acid production, for instance, and exerting probiotic health-promoting effects to the vertebrate host [79,80,81]. In line with this, carvacrol application to chicken broilers was shown to delay intestinal colonization with *Campylobacter* species due to changes in the commensal gut microbiota composition towards a higher abundance of distinct probiotic bacterial species [33]. Of note, carvacrol has been approved as an animal food supplement by the European Union in order to reduce the abundance of *Campylobacter* in livestock and meat production [82]. Furthermore, the Federal Drug Administration (FDA) has considered synthetic carvacrol as a safe chemical compound that is used as preservative in the food industry [83].

## 5. Conclusions

In conclusion, the results of our preclinical placebo-controlled intervention study provide evidence that the therapeutic application of carvacrol dampens intestinal and extra-intestinal inflammatory responses during acute campylobacteriosis in IL-10^−/−^ mice harboring a human gut microbiota. Given the worldwide threat of antimicrobial resistance, this compound may be considered as a potent antibiotic-independent treatment option for acute campylobacteriosis in immune-compromised humans and, additionally, for the reduction in *C. jejuni* colonization in livestock, and may therefore improve food safety.

## Data Availability

The corresponding author provides data upon request.

## Supplementary Materials

The following supporting information can be downloaded at: https://www.mdpi.com/article/10.3390/biom13020320/s1, Figure S1: Microbiota composition of human fecal donor suspensions; Figure S2: Fecal microbiota changes upon carvacrol treatment of *C. jejuni*-infected IL-10^−/−^ mice harboring a human gut microbiota.
