# Supplementary material for: Therapeutic Oral Application of Carvacrol Alleviates Acute Campylobacteriosis in Mice Harboring a Human Gut Microbiota
Source: Biomolecules. 2023 Feb 8;13(2):320. doi: 10.3390/biom13020320 (PMC9953218; doi:10.3390/biom13020320)
Supplement: Supplementary file 1 [file biomolecules-13-00320-s001.zip › biomolecules-2190211-supplementary.pdf]

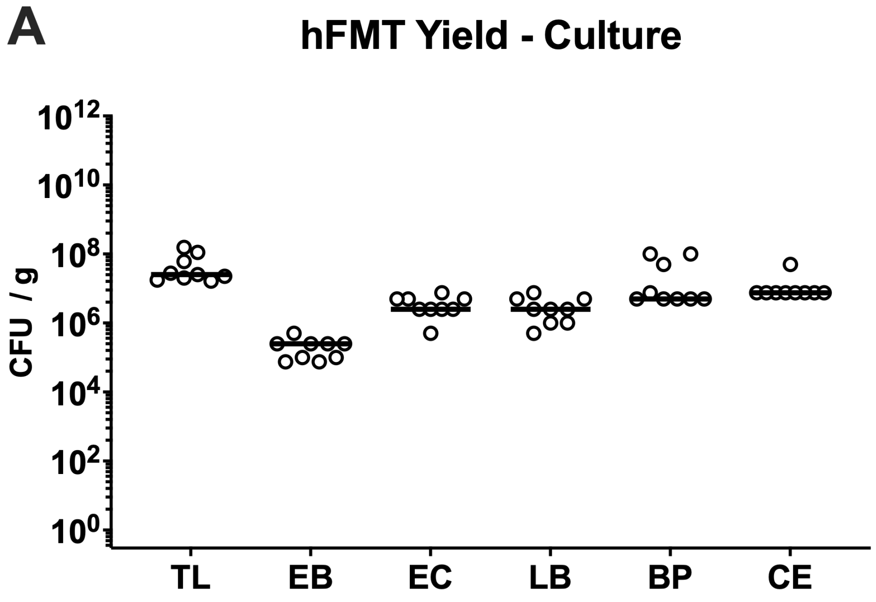

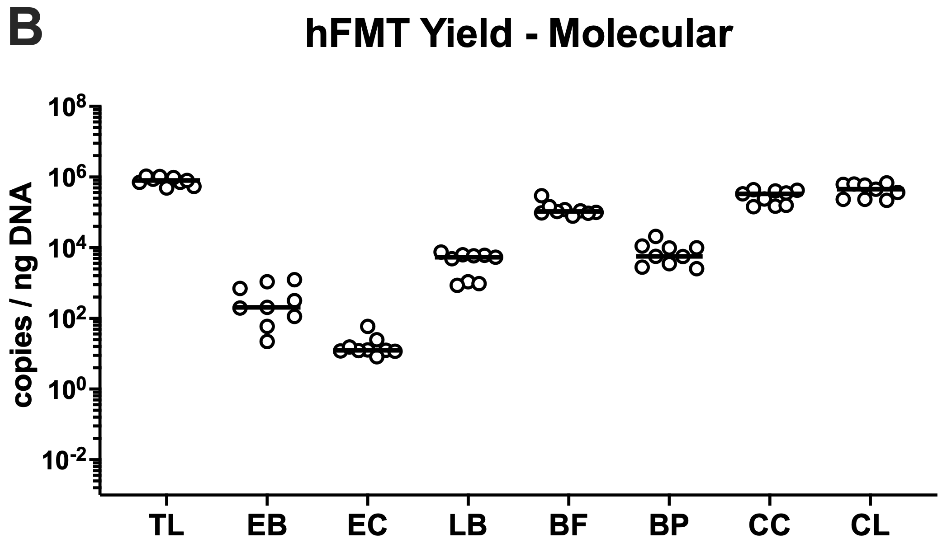

Supplementary Figure S1. Microbiota composition of human fecal donor suspensions. Secondary abiotic mice were subjected to human fecal microbiota transplantion (hFMT) on three consecutive days starting a week prior infection (i.e., days -7, -6, -5). The human fecal microbiota composition was quantitatively surveyed in respective donor suspensions by both, **(A)** culture and **(B)** culture-independent, molecular methods. Bacterial loads are expressed as colony-forming units per gram (CFU / g) and gene copies per ng DNA, respectively. Data pooled from three experiments and medians are shown. TL, total load; EB, enterobacteria; EC, enterococci; LB, lactobacilli; BB, bifidobacteria; BP, *Bacteroides/Prevotella* species; CE, *Clostridium/Eubacterium* species; CC, *Clostridium coccoides* group; CL, *Clostridium leptum* group.

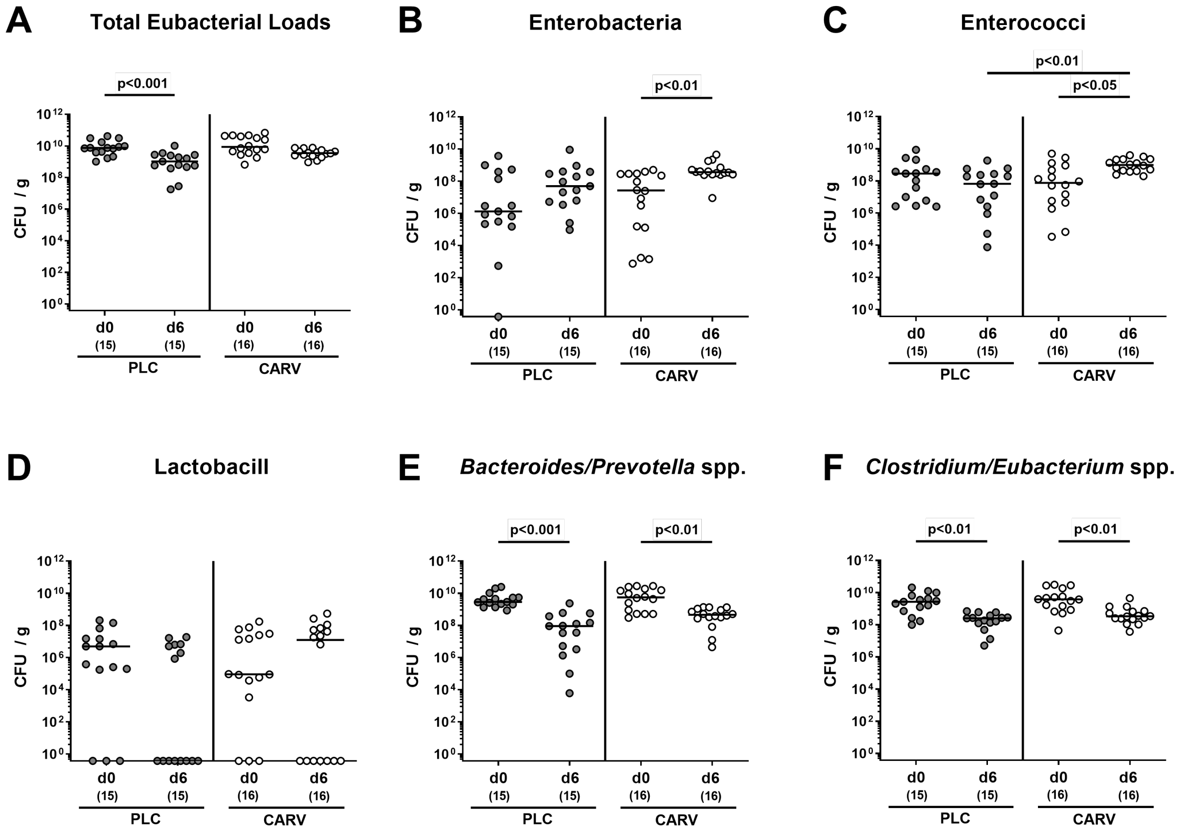

Supplementary Figure S2. Fecal microbiota changes upon carvacrol treatment of *C. jejuni* infected IL-10$^{-/-}$ mice harboring a human gut microbiota.

IL-10$^{-/-}$ mice harboring a human gut microbiota were perorally infected with *C. jejuni* on day (d) 0 and d1. Starting on d2 post-infection (p.i.), mice were treated with either synthetic carvacrol (CARV, white circles) or placebo (PLC, grey circles) via the drinking water until d6 p.i. Before (d0) and upon sacrifice 6 days after infection, the fecal microbiota composition was quantitatively surveyed by culture and the respective bacterial numbers expressed as colony-forming units per gram (CFU / g). Data pooled from three experiments, medians, numbers of included mice (in parentheses), and significance levels (p values) as determined by the Kruskal-Wallis test and Dunn's post-correction are shown.
